# Conjugated Polymer-Photosensitizers for Cancer Photodynamic Therapy and Their Multimodal Treatment Strategies

**DOI:** 10.3390/polym17091258

**Published:** 2025-05-05

**Authors:** Zhengqing Cheng, Qiuting Ye, Jieling Lao, Xiyu Liu, Pan Wu

**Affiliations:** 1 State Key Laboratory of Targeting Oncology, National Center for International Research of Bio-Targeting Theranostics, Guangxi Key Laboratory of Bio-Targeting Theranostics, Collaborative Innovation Center for Targeting Tumor Diagnosis and Therapy, Guangxi Medical University, Nanning 530021, China; zhengqing1108@163.com (Z.C.); ye_qiuting@163.com (Q.Y.); 13107379400@163.com (J.L.); 2 School of Pharmacy, Guangxi Medical University, Nanning 530021, China

**Keywords:** conjugated polymers, photosensitizer, tumor, photodynamic therapy, multimodal therapy

## Abstract

Conjugated polymers (CPs) have emerged as promising candidates for photodynamic therapy (PDT) in cancer treatment due to their high fluorescence quantum yield, excellent photostability, and remarkable reactive oxygen species (ROS) generation capability. This review systematically summarizes molecular design strategies to augment CP photosensitivity efficiency, including: (1) constructing donor–acceptor (D-A) alternating structures, (2) incorporating aggregation-induced emission (AIE) moieties, (3) employing heavy-atom effects, and (4) designing hyperbranched architectures. In addition, considering the limitations of monotherapy like tumor heterogeneity, we will further discuss the synergistic treatment strategies of CP-mediated PDT in combination with other therapeutic modalities, including photothermal therapy (PTT)-PDT, immunotherapy-PDT, chemotherapy-PDT, Chemiluminescence (CL)-PDT, diagnostic technology-PDT, and chemodynamic therapy (CDT)-PDT. These multimodal approaches leverage complementary mechanisms to achieve enhanced tumor eradication efficacy.

## 1. Introduction

Cancer remains a leading cause of death worldwide and poses major medical challenges due to its uncontrolled growth, aggressive spread, and resistance to complete eradication [1,2,3,4]. Traditional mainstream clinical treatments such as surgery, radiotherapy, and chemotherapy can partially suppress cancer progression but are accompanied by severe tissue damage, systemic toxicity, and drug resistance in cancer cells, greatly limiting their therapeutic efficacy and applicability [5,6,7,8]. Therefore, exploring highly efficient and patient-compliant new therapies is crucial for cancer treatment.

In recent years, photodynamic therapy (PDT) has emerged as an alternative to conventional cancer treatments due to its minimally invasive nature, spatiotemporal precision, and low side effects [9,10,11,12]. PDT is a therapeutic approach based on photosensitizer (PS), oxygen, and light irradiation. The core mechanism involves the absorption of photon energy by the PS under specific wavelengths, exciting it from the ground state (singlet state, S_0_) to the excited singlet state (S_1_). Subsequently, through intersystem crossing (ISC), it transitions to the longer-lived triplet excited state (T_1_), generating reactive oxygen species (ROS) via two distinct pathways. In the Type I pathway, the excited PS undergoes electron or proton transfer with nearby endogenous substrates in tumor cells, producing cytotoxic radicals such as superoxide radicals (•O_2_^−^), hydrogen peroxide (H_2_O_2_), and hydroxyl radicals (•OH). In the Type II pathway, energy transfer between the excited PS and surrounding oxygen converts ground-state oxygen into cytotoxic singlet oxygen (^1^O_2_) [13,14,15]. These ROS disrupt the structure and function of biomolecules, leading to efficient tumor cell destruction [16,17]. Among these components, the PS is the core determinant of PDT efficacy and has been a major research focus.

Conjugated polymers (CPs) were initially widely used in optoelectronic devices and photovoltaics due to their excellent conductivity and light absorption properties [18,19,20,21]. Further studies revealed that the π-conjugated structure of CPs not only excels in optoelectronic applications but also generates ROS through energy or electron transfer—a characteristic highly aligned with the core mechanism of PDT [22,23]. This discovery expanded their application in biotherapy, establishing CPs as promising PS for PDT. Currently, CPs have been applied in the treatment of many biological fields, particularly in cancer treatment, where CP-based PDT effectively kills tumor cells [24,25,26,27,28].

Although CP-mediated PDT shows great potential, it faces common challenges associated with PS, such as the short diffusion radius and limited lifetime of ROS, as well as poor tissue penetration of light, which restricts its application in tumor therapy [29,30,31]. To overcome these limitations, researchers have explored various strategies. On the one hand, efforts have focused on the design of conjugated polymers with highly efficient photosensitizing properties to enhance ROS generation efficiency, thereby improving the therapeutic outcome of PDT at its root [32]. On the other hand, from the perspective of optimizing overall treatment strategies, functional modifications have been applied to CPs by integrating them with photothermal conversion, immune regulation, chemical catalysis, and other functional modules to achieve multimodal synergistic therapy [33,34,35]. This combined therapeutic approach is expected to address the efficacy limitations of CP-mediated single-mode PDT, significantly improving tumor treatment outcomes.

This review first summarizes molecular design strategies to improve CPs’ photodynamic efficacy: constructing donor–acceptor (D-A) structures to promote intramolecular charge transfer, enhancing exciton dissociation and ISC efficiency; introducing aggregation-induced emission (AIE) groups to overcome aggregation-caused quenching (ACQ) and improve light-harvesting capability; utilizing the heavy-atom effect to strengthen spin-orbit coupling and accelerate ISC kinetics; and designing hyperbranched structures to increase active site exposure and optimize exciton transport pathways. These molecular engineering approaches systematically regulate the excited-state behavior of CPs, providing a reference for developing high-performance CP-based photosensitizers.

**Scheme 1:**
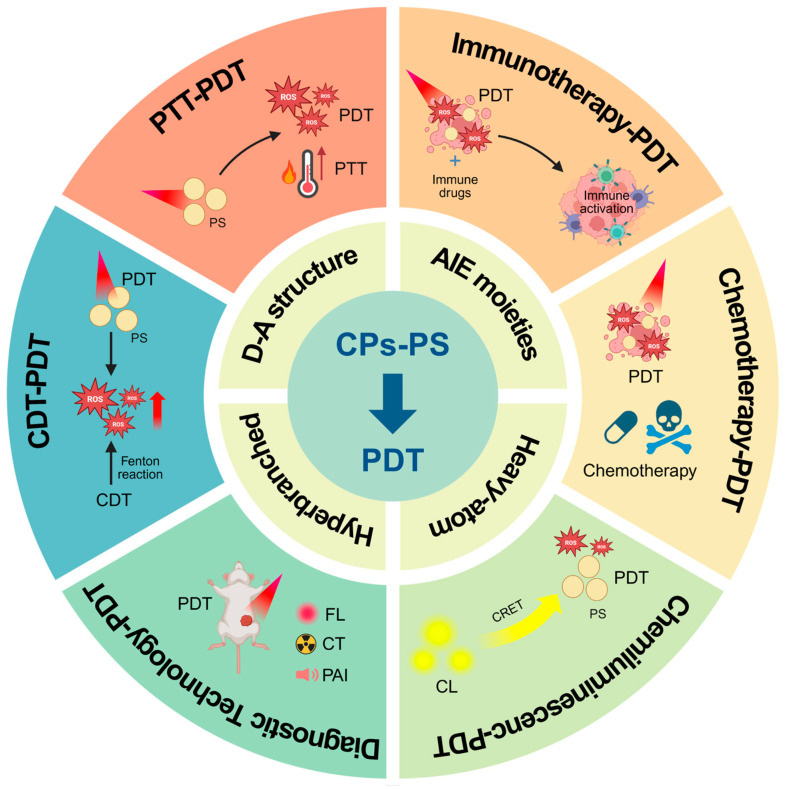
Schematic of molecular design strategies for enhancing photodynamic effects in conjugated polymers and their multimodal therapeutic integrations.

Furthermore, addressing the limitations of monotherapy in cancer treatment, we explore CP-mediated PDT combined with other therapeutic strategies to enhance antitumor efficacy: Integration with photothermal therapy (PTT), where light-generated heat further kills tumor cells; combination with immunotherapy, activating the immune system for systemic tumor cell elimination; synergy with chemotherapy, improving tumor cell killing efficiency while reducing drug resistance; coupling with Chemiluminescence (CL), overcoming the limited penetration depth of traditional PDT light sources; combination with imaging techniques, enabling real-time treatment monitoring; and integration with chemodynamic therapy (CDT), leveraging chemically generated reactive species to enhance therapeutic outcomes. This multimodal cancer treatment approach aligns with the current multidisciplinary paradigm in oncology, enabling efficient tumor ablation and providing insights for designing next-generation therapeutic platforms (Scheme 1).

## 2. Design Strategy and Performance Optimization

Conjugated polymers (CPs) are a class of high-molecular-weight organic compounds composed of repeating conjugated structural units featuring extended π-conjugated delocalized electronic structures. Their unique electron delocalization characteristics enable charge carriers to migrate freely along the molecular chain, exhibiting semiconductor-like behavior. The most attractive feature of these materials lies in their highly tunable molecular design: by precisely selecting the type, arrangement, and topology of conjugated units, key optoelectronic parameters such as bandgap, light absorption properties, and charge carrier mobility can be precisely modulated [36,37,38]. In recent years, conjugated polymers have demonstrated immense potential in photodynamic therapy (PDT) due to their exceptional photostability, broad and strong absorption characteristics, and efficient reactive oxygen species (ROS) generation [22].

In PDT, the efficiency of ROS generation directly determines therapeutic efficacy, with the key to enhancing ROS yield lying in optimizing the intersystem crossing (ISC) process of the photosensitizer (PS) and prolonging the triplet-state lifetime. The ISC process involves a spin-flip transition from the singlet excited state (S₁) to the triplet excited state (T₁). A higher ISC efficiency means more molecules can populate the T₁ state, thereby providing a richer pool of reactive substrates for subsequent ROS generation. Meanwhile, the triplet-state lifetime determines the duration of T₁-state molecules remaining excited—the longer the lifetime, the higher the probability of energy transfer between triplet-state molecules and oxygen, ultimately leading to greater ROS production [39,40]. Thus, improving ISC efficiency and extending triplet-state lifetime are crucial for enhancing the PDT performance of conjugated polymers. From molecular design to functional output, the performance optimization of conjugated polymers in PDT follows a progressive logic of “structural design → photophysical modulation → efficient ROS generation”. Through rational selection of conjugated units and optimization of topological structures, the energy-level structure and excited-state dynamics of the material can be precisely regulated, thereby enhancing ISC efficiency and stabilizing the triplet state. This ultimately enables high-concentration, long-lived T₁-state molecules to fully interact with oxygen, significantly boosting ROS yield and intensifying oxidative damage to tumor cells, thereby markedly improving PDT efficacy. Based on this mechanism, this chapter will systematically explore design strategies to enhance the photosensitizing performance of conjugated polymers from the perspective of molecular engineering.

### 2.1. D-A Structure Engineering

Donor–acceptor (D-A) type-conjugated polymers represent a class of high-performance materials constructed through alternating connections between electron-donating (D) and electron-accepting (A) units. In these structures, the donor component provides electron-rich characteristics that facilitate hole transport, while the acceptor possesses electron-deficient properties that promote electron transport. By carefully selecting different D/A unit combinations and arranging them in an alternating fashion, the intramolecular charge transfer (ICT) effect can be significantly amplified. This effect induces charge redistribution within the molecule, where electrons transfer from the donor to the acceptor moiety, effectively reducing the material’s bandgap and enhancing the ISC efficiency from the lowest excited singlet state to the lowest triplet state. 

**Figure 1:**
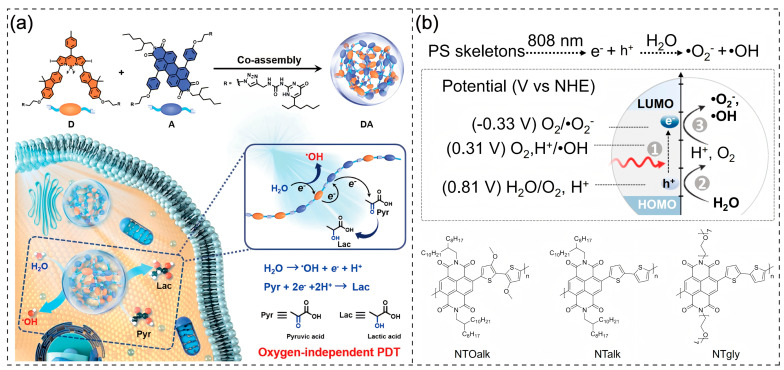
(**a**) Schematic illustration of the preparation of DA, as well as photoinduced generation of •OH and reduction of pyruvic acid [45]. Copyright © 2023, American Chemical Society. (**b**) The detailed molecule skeleton design strategy toward proposed organic photosensitizers with low bandgap and appropriate redox potential to sensitize H_2_O into •O_2_^−^ and •OH to achieve 808 nm photoexcitation, •O_2_^−^ independent •O_2_^−^ and •OH production, and the chemical structures of NTOalk, NTalk, and NTgly [61]. Copyright 2024, Springer Nature.

Moreover, this structural modification extends the T₁ lifetime, creating favorable conditions for generating more ROS in photodynamic therapy, thereby improving the overall therapeutic efficacy [41,42,43,44]. Yang et al. designed a supramolecular polymeric PS by co-assembling fluorine-substituted BODIPY (as the donor, D) with perylene diimide (as the acceptor, A) for •OH generation via water oxidation. The D-A assembly exhibited a strong photocurrent, whereas negligible photocurrent was observed for isolated D or A components. The charge transfer resistance of the D-A system was substantially lower than that of individual D or A, indicating efficient charge separation and accelerated charge migration rates, which synergistically amplified the ICT effect. Laser flash photolysis measurements revealed a dramatic increase in triplet-state lifetime from 1.16 μs (isolated D) to 15.78 μs (D-A assembly). A longer triplet-state lifetime means that the excited-state molecules have more time to participate in subsequent reactions, enabling more efficient generation of highly cytotoxic •OH. These results confirm that the DA co-assembled polymer PS exhibits significantly enhanced photophysical and photochemical properties compared to its individual components, demonstrating superior potential for photodynamic therapy applications [45] (Figure 1a).

Extensive research and clinical practice have confirmed that traditional Type II photosensitizers generate ^1^O_2_ by transferring T_1_ energy to molecular oxygen, exhibiting high oxygen dependency that severely compromises the efficacy of PDT in hypoxic tumor microenvironment (TEM) [46,47]. In contrast, the Type I photodynamic mechanism enables the generation of radicals via electron transfer between excited-state PS and endogenous biomolecules (e.g., proteins, nucleic acids, or lipids) even under severe hypoxia, thereby overcoming oxygen-dependent limitations. Since the redox potential of PS directly determines its capacity to participate in electron transfer, the redox properties of PS can be modulated to satisfy the thermodynamic requirements for photosensitive reactions with endogenous biological substrates [48,49,50]. CPs, characterized by extended π-conjugated backbones, facilitate electron delocalization and promote ICT, which consequently regulates their redox potentials [51,52,53]. In D-A structured CPs, ICT from donor to acceptor units enhances the oxidation tendency of donors and the reduction tendency of acceptors, endowing the molecules with sufficiently high oxidation potentials and sufficiently low reduction potentials [54]. This synergy fulfills the thermodynamic criteria for oxygen-independent Type I photodynamic pathways, offering a novel strategy to circumvent hypoxia-limited PDT in cancer treatment.

In Type I photodynamic pathways, photosensitizers require photosensitive reactions with endogenous substrates to exert their therapeutic effects. However, endogenous macromolecular substrates like glutathione (GSH) and nicotinamide adenine dinucleotide (NADH) are unevenly distributed and present in limited quantities within organisms, significantly constraining their photosensitive interactions with photosensitizers [55,56,57]. Fortunately, water is abundantly available in biological systems. Therefore, designing photosensitizers that utilize water as the photosensitive substrate presents a viable strategy to enhance PDT efficacy [58,59,60]. As designed by Yang’s research group, the D-A type-conjugated polymer exhibits an oxidation potential higher than E_OH_^−^_/•OH_, enabling it to oxidize water and generate •OH upon photoexcitation. In vitro experimental results demonstrate that this PS can stably produce •OH under light irradiation, regardless of normoxic or hypoxic conditions [45]. In another related study, based on the frontier molecular orbital theory, Yu et al. proposed coupling electron donors with low Highest Occupied Molecular Orbital (HOMO) levels and electron acceptors to develop near-infrared-excited photosensitizers with high redox potentials. This design enables the use of water molecules as photosensitive substrates, achieving oxygen-independent photodynamic therapy. Through systematic screening, they selected naphthalenediimide as the electron acceptor and bithiophene as the electron donor, incorporating different side chains to design three distinct CPs: NTOalk, NTalk, and NTgly. 

In order to illustrate the relationship between redox potential and the conversion of water to ROS by photosensitizers from a thermodynamic point of view, the redox potentials of the polymers NTOalk, NTalk, and NTgly were measured using cyclic voltammetry, in which the oxidation potentials of NTalk and NTgly were 1.12 V and 1.15 V, respectively, which were higher than 0.81 V vs. NHE (H_2_O + 4h^+^ → O_2_ + 4H^+^) oxidation potential, which satisfies the thermodynamic condition that photosensitizers can oxidize H_2_O to O_2_ after being photoexcited, whereas the oxidation potential of NTOalk is 0.59 V less than 0.81 V, which does not have this condition.

In addition, the reduction potentials of NTOalk, NTalk, and NTgly are −0.72 V, −0.66 V, and −0.64 V, respectively, which are lower than −0.33 V vs. NHE (O_2_ + e^−^ → •O_2_^−^) or 0.31 V vs. NHE (O_2_ + 3H^+^ + 3e^−^ → •OH +H_2_O) reduction potentials, suggesting that all three can reduce O_2_ to •O_2_^−^, •OH. The solution-level assay results also indicate that the photoexcited NTOalk has the ability to convert O_2_ to •OH only in the O_2_ to •O_2_^−^; •OH under normoxia, while photoexcited NTalk and NTgly can still produce •O_2_^−^ even in the anoxic environment. •OH and the isotopic detection results proved that the oxygen molecules in the generated •O_2_^−^ and •OH all originated from H_2_O [61] (Figure 1b).

In summary, the distinctive donor–acceptor alternating structure of D-A type-conjugated polymers can amplify intramolecular charge transfer effects, enhance intersystem crossing efficiency, prolong triplet-state lifetime, and increase reactive oxygen species generation. Moreover, the rational design of a donor–acceptor unit assemblies based on frontier orbital theory enables effective regulation of the conjugated polymer’s orbital energy levels, thereby satisfying the thermodynamic requirements of Type I photodynamic pathways. This characteristic allows the material to efficiently excite endogenous substances for reactive oxygen species production under both normoxic and hypoxic conditions. Most importantly, through a targeted design utilizing water molecules, which are abundant in biological systems as substrates, these D-A type-conjugated polymer photosensitizers can achieve substantial enhancement of reactive oxygen species yield in the hypoxic tumor microenvironment, significantly improving the efficacy of photodynamic therapy.

### 2.2. Incorporation of AIE Moieties

The planar aromatic structure of CPs tends to induce aggregation-caused quenching (ACQ) in the aggregated state, which fundamentally arises from intermolecular π–π stacking that promotes non-radiative decay of excited-state energy through pathways such as intramolecular vibrations and thermal dissipation, significantly reducing fluorescence intensity and photosensitization efficiency [62,63,64]. This phenomenon directly compromises the generation efficiency of ROS in PDT, limiting its clinical application potential. Fortunately, aggregation-induced emission (AIE) molecules offer an innovative strategy to address this challenge. Most AIE molecules possess highly distorted propeller-shaped structures, a unique conformation that endows them with dynamic, responsive characteristics. In dilute solutions, AIE molecules dissipate energy through non-radiative transitions, whereas in the aggregated state, further conformational distortion inhibits π–π stacking and restricts intramolecular motions, thereby enabling energy release through radiative transitions or ROS generation [65,66,67]. By incorporating AIE molecules into the main chain or side chains of CPs, the resulting AIE-CPs composite materials not only retain the strong fluorescence emission and efficient ROS generation capabilities of AIE in the aggregated state but also benefit from the broad spectral absorption and exciton migration properties conferred by the π-conjugated skeleton of CPs.

**Figure 2:**
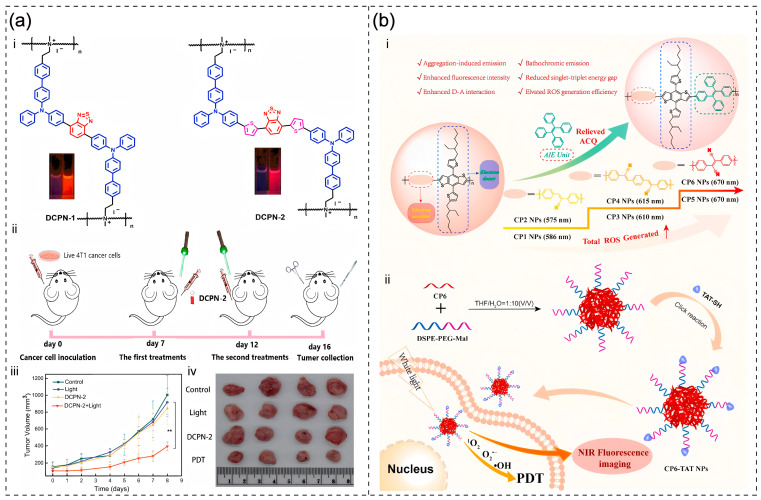
(**a**) (**i**) Chemical structures of DCPN-1 and DCPN-2. (**ii**) Schematic diagram of nude mice for photodynamic therapy. (**iii**) Tumor growth profiles of nude mice in different groups. (DCPN-2: 1 mg mL^−1^ based on PBS, 50 μL per mice. Values represent means ± SD (n = 4), ** *p* < 0.01). (**iv**) The photographs of tumors collected from different groups of nude mice at day 10 posttreatment [68]. Copyright © 2024 Wiley-VCH GmbH. (**b**) Graphic illustration of (**i**) molecular engineering of CPx NPs and (**ii**) the preparation procedure of CP6-TAT NPs and their application in NIR fluorescence imaging and PDT [69]. Copyright © 2022 Elsevier Ltd. All rights reserved.

Cong et al. successfully prepared two NIR AIE cationic polymers, DCPN-1 and DCPN-2, by introducing triphenylamine with AIE properties into the backbone and using ring-opening polymerization. Due to the presence of AIE-emitting groups, the polymers showed enhanced fluorescence emission and facilitated the conversion of O_2_ to ^1^O_2_ due to the restricted intramolecular motion in the aggregated state, and DCPN-2 showed a higher ^1^O_2_ yield under white light irradiation than that in commercial photosensitizers Ce6. In in vivo experiments, the “PDT (DCPN-2 + light)” group inhibited the growth of tumors in 4T1 ruffled nude mice, with a 55% reduction in tumor weight compared to the control group [68] (Figure 2a). Zhang et al. introduced tetraphenylethylene (TPE) into the D-A skeleton to alleviate the ACQ effect of CPs and enhance their performance in the aggregated state. In the solution state, the spatial proximity of D-A promotes intermolecular close-packing, which leads to a decrease in fluorescence intensity, but the introduction of TPE, which has the property of AIE, successfully inhibits the intermolecular close-packing, greatly reduces the energy loss, and enables efficient luminescence and ROS generation in the aggregated state of the emission spectrum while enhancing the ROS generation efficiency, indirectly optimizing the ROS generation ability of CPs. Correspondingly, the CP6 NPs in the present paper have a higher ^1^O_2_ yield due to their suitable structure and AIE-related properties, which enables them to kill cancer cells more effectively [69] (Figure 2b).

### 2.3. Heavy-Atom Effects

**Figure 3:**
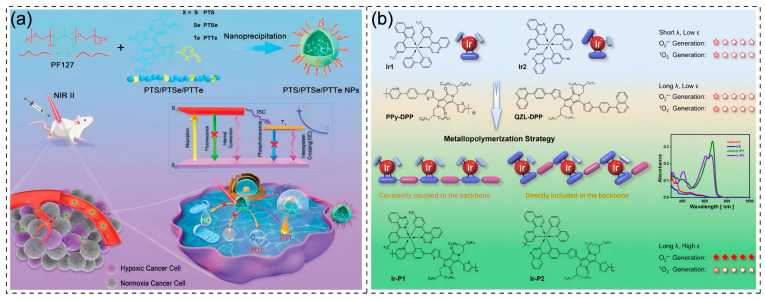
(**a**) Schematic illustration of the preparation of PTS, PTSe, and PTTe NPs and used as NIR-II type-I PDT/PTT photosensitizers under hypoxic conditions [73]. Copyright © 2022 Elsevier Inc. All rights reserved. (**b**) Metallopolymerization to explore high-performance Type-I photosensitizers with strong absorption in the long-wavelength region [79]. Copyright 2024, Springer Nature.

Heavy atoms such as iodine, bromine, iridium, and platinum possess large atomic numbers and strong spin-orbit coupling (SOC) effects. In PDT, the triplet excited state serves as the critical intermediate for ROS generation. The introduction of heavy atoms enables robust SOC to effectively promote ISC from S₁ to T₁ states, allowing more molecules to populate triplet excited states and thereby establishing the foundation for efficient ROS production [68,69,70,71,72]. Wen et al. incorporated heavy atoms (S, Se, Te) into the backbone of CPs, developing three CPs (PTS, PTSe, and PTTe) as NIR-II-responsive Type I photosensitizers for tumor treatment. Remarkable performance variations were observed due to the substantial atomic size differences from S to Te atoms. Notably, the spin-orbit coupling constant ξ (S_1_, T_n_) increased by nearly one order of magnitude from S to Te atoms, dramatically accelerating the ISC process. The energy gap (ΔEST) between the S_1_ state and the neighboring T_3_ state is calculated based on the density-functional theory to decrease gradually from 0.36 eV for PTS, 0.35 eV for PTSe to 0.33 eV for PTTe, which leads to a significant increase in the ISC rate constant (kISC), corresponding to the kISC of 2.19 × 10^4^, 1.80 × 10^5^ and 3.55 × 10^5^ s^−1^ for PTS, PTSe, and PTTe. In the ROS yield detection experiments, the nanoparticles of PTTe showed the most outstanding performance, which strongly proves that the heavy atom effect significantly enhances the generation efficiency of the triplet excited state in the photodynamic process and improves the photodynamic effect [73] (Figure 3a).

Many metal coordination complexes suffer from limited absorption wavelengths and low molar extinction coefficients due to insufficient ligand conjugation. Their absorption typically resides in ultraviolet or short-wavelength visible regions (e.g., some Ir(III) complexes with absorption below 600 nm and Ru complexes with absorption peaks around 450 nm), thus restricting their application in solid tumor therapy [74,75,76]. The poor light-harvesting capability further compromises ROS generation efficiency under specific wavelengths. In contrast, CPs featuring extended π-conjugated systems enable tunable absorption and emission characteristics through structural modifications. Integrating metal complexes into such π-conjugated frameworks can broaden absorption spectra and enhance light absorption [77,78]. Zhang et al. directly embedded an Ir(III) complex with Type I ROS generation capability into the polymer backbone, developing Ir-P2 as a high-performance Type I photosensitizer with strong deep-red absorption. Under 680 nm laser irradiation, Ir-P2 demonstrated over 80-fold higher ROS generation compared to its iridium-free counterpart, PPy-DPP. Notably, Ir-P2 exhibited a red shift to 700 nm in the absorption spectrum relative to PPy-DPP. This strategic combination of heavy atoms and CPs provides a promising approach for developing photosensitizers with both efficient photosensitization and strong NIR absorption, paving the way for treating deep-seated solid tumors [79] (Figure 3b).

In addition, bare heavy atoms exhibit strong toxicity, which limits their application in biological systems. Liu et al. designed a selenium-containing small-molecule photosensitizer, Secy7. Although Secy7 demonstrated excellent photodynamic performance under light irradiation, it displayed significant selenium-derived heavy-atom toxicity in dark conditions [80]. Fortunately, incorporating heavy atoms into CPs can mitigate these issues: the planar conjugated backbones of CPs restrict the migration and leaching of heavy atoms, while chemical covalent conjugation between the two components prevents the exposure of highly toxic bare heavy atoms, thereby enhancing biocompatibility. The heavy-atom-incorporated CPs developed by the Wen and Zhang research groups, as mentioned above, showed no apparent dark toxicity [73,79].

### 2.4. Hyperbranched

Hyperbranched CPs have a three-dimensional dendritic structure, which endows them with unique properties. Structurally, it possesses a large number of terminal groups and internal cavities, and this structure gives it a good spatial separation effect, which reduces intramolecular interactions and energy loss, facilitates the transfer of electrons between different energy levels, and thus increases the number of ISC channels [81,82]. In addition, in photodynamic therapy studies, the dendritic structure exhibited by hyperbranched CPs as photosensitizers resulted in the formation of a porous structure inside the polymer, which better-dispersed oxygen and increased the contact area with oxygen, thus improving the ROS yield [83].

Cheng et al. synthesized three types of CP photosensitizers by different assemblies of triphenylamine and anthraquinone, which were the main-chain polymer MP, the side-chain polymer SP and hyperbranched polymer HP, and the electronic structures of the model compounds of the monomer small molecules as well as the polymers MP, SP, and HP were calculated by density functional theory (DFT) and time-containing density functional theory (TD-DFT), and the results demonstrated that the energy level differences of the compounds after conjugate polymerization became smaller, resulting in more ISC channels. In addition, the difference between the different energy levels of the model compounds corresponding to hyperbranched polymers is much smaller than that of the conventional main-chain polymers, and this feature of the energy level distribution allows the hyperbranched polymers to have more ISC channels and produce ROS more efficiently. The photosensitization efficiencies corresponding to the hyperbranched polymer HP are much higher than that of the main-chain polymers MP and the side-chain polymers SP at the solution level, with the •O_2_^−^ yield of the hyperbranched polymer HP being higher than that of MP and the side-chain polymers SP. The •O_2_^−^ yield was 2.1 times higher than that of MP and 9.7 times higher than that of SP; the ^1^O_2_ generation efficiency was 1.8 times higher than that of MP and 2.5 times higher than that of SP; and •OH could not be generated by MP and SP but could be generated by HP.

**Figure 4:**
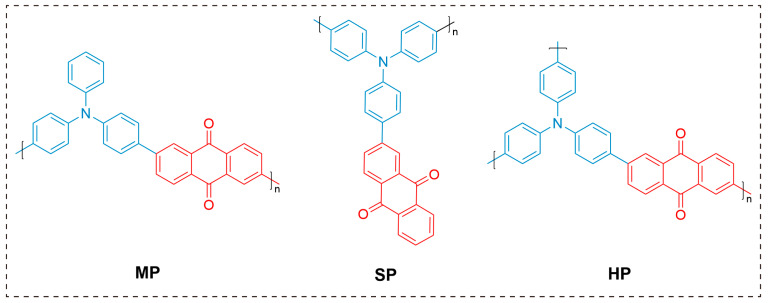
The chemical structures of main-chain polymer MP, side-chain polymer SP, and hyperbranched polymer HP [84]. Adapted from 2022, The Royal Society of Chemistry.

Furthermore, the generation of ROS relies on the interaction between photosensitizers and oxygen. Therefore, the higher ROS production efficiency of hyperbranched polymers indirectly demonstrates that their hyperbranched architecture enables more effective oxygen contact. In cellular and animal-level photodynamic therapy experiments, the “HPt NPs + Laser” group exhibited significantly elevated intracellular ROS levels and markedly suppressed tumor growth upon laser irradiation. These results confirm the unique advantages of hyperbranched conjugated polymers as high-performance photosensitizers [84] (Figure 4)

## 3. Multimodal Cancer Therapy Based on Conjugated Polymers

Conjugated polymers (CPs) have attracted considerable interest in photosensitizer (PS) development owing to their remarkable light absorption capacity, tunable electronic structures, and excellent biocompatibility [85,86]. Nevertheless, photodynamic therapy (PDT), which depends exclusively on reactive oxygen species (ROS)-mediated cytotoxic mechanisms, encounters multiple challenges in practical applications. The limited tissue penetration depth of excitation light, particularly visible light, makes it difficult to effectively treat deep-seated tumors or complex anatomical structures [31,87]. Moreover, the short diffusion radius of ROS (e.g., ^1^O_2_ with a range of merely ~20 nm) leads to insufficient eradication of tumor cells located distal to the photosensitizers [29,30]. Additionally, the immunosuppressive tumor microenvironment substantially diminishes the immunogenic cell death (ICD) effect induced by PDT, thereby compromising therapeutic outcomes [88]. To address these limitations, researchers have developed multimodal therapeutic strategies that combine different treatment modalities to achieve spatial and functional complementarity, resulting in synergistic enhancement of antitumor efficacy. This chapter will concentrate on cutting-edge representative applications of CP-mediated PDT integrated with various therapeutic approaches, providing comprehensive insights into their combination with photothermal therapy (PTT), immunotherapy, chemotherapy, chemiluminescent light sources, diagnostic techniques, and chemodynamic therapy (CDT) for synergistic treatment.

### 3.1. PTT-PDT

Photothermal therapy (PTT) depends on photothermal conversion materials such as polydopamine, single-walled carbon nanotubes, or metal nanoparticles to efficiently convert absorbed light energy into thermal energy. This thermal ablation effect triggers protein denaturation, cell membrane rupture, and vascular system collapse, thereby achieving physical tumor destruction [89,90,91]. In contrast, PDT generates cytotoxic ROS through photosensitive units under specific wavelength excitation, initiating oxidative stress responses such as lipid peroxidation, mitochondrial damage, and deoxyribonucleic acid (DNA) fragmentation [16].

**Figure 5:**
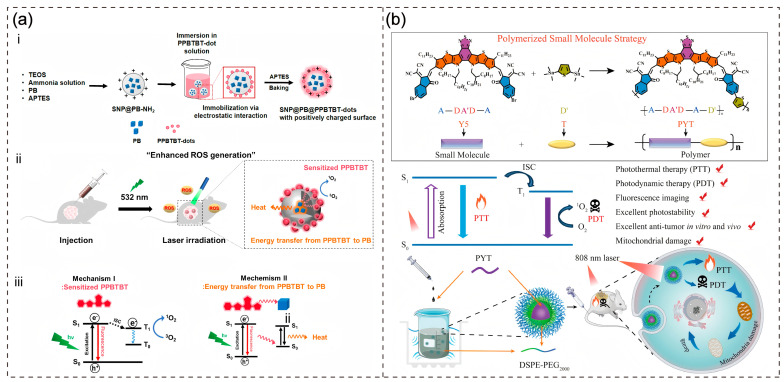
(**a**) (**i**) Schematic Illustration of Fabrication of SNP@PB@PPBTBT dots. (**ii**) Schematic illustration of the enhanced generation of ROS by SNP@PB@PPBTBT dots-in-tumor-bearing mice under 532 nm laser illumination for effective PDT. (**iii**) Schematic illustration of the generation of ROS via the triplet state of PPBTBT and the energy transfer from sensitized PPBTBT to photothermal PB to generate heat [93]. Copyright © 2023, American Chemical Society. (**b**) PYT enabled by polymerized small molecule strategy for tumor photothermal and photodynamic therapy [94]. Copyright 2023, Springer Nature.

These two modalities exhibit marked differences in their mechanisms of action, and their combined application forms a “thermo-oxidative” dual-modal synergistic therapeutic system. On the one hand, PTT-induced tumor vascular dilation and accelerated blood flow can improve oxygen partial pressure in the tumor microenvironment (TEM), alleviating the oxygen-dependent limitation of PDT. On the other hand, PDT-generated ROS can attenuate the thermal tolerance of tumor cells, enhancing the thermal sensitivity threshold of PTT [92]. Jo et al. successfully synthesized SNP@PB@PPBTBT dots with dual PTT/PDT effects by embedding photothermally active Prussian blue (PB) into silica nanoparticles (SNPs). The surface of SNPs was functionalized with amine groups to confer a positive charge, followed by electrostatic assembly with the negatively charged photodynamic agent PPBTBT. In this composite, PB serves as a photothermal agent, where the absorbed light energy generates heat to induce tumor thermal ablation. Simultaneously, the temperature elevation accelerates the reaction kinetics between CPs and oxygen, thereby promoting enhanced ROS generation by CPs. Experimental data revealed that, under laser irradiation, the PB-containing composite achieved a temperature rise to 45 °C and a 35% increase in ROS production compared to PB-free controls. In cellular assays, tumor cells treated with SNP@PB@PPBTBT dots exhibited significantly elevated intracellular ROS levels. Animal studies further demonstrated that tumors subjected to this combinatorial therapy exhibited markedly slower growth rates than those treated with monotherapy, confirming the system’s efficacy in suppressing tumor progression [93] (Figure 5a).

Notably, the broad-spectrum absorption characteristics of CPs enable simultaneous responsiveness to light sources of distinct wavelengths, achieving spatiotemporally synchronized activation of photothermal and photodynamic effects. This dual mechanism—combining physical and thermal ablation with chemical oxidative damage—not only expands the spatial coverage of tumor destruction but also overcomes tumor heterogeneity through multi-target engagement, thereby significantly suppressing therapeutic resistance [95]. Xie et al. synthesized CPs PYT by integrating the A-DA′D-A-structured small molecule Y5, characterized by a narrow bandgap and strong light absorption, with thiophene units via Stille cross-coupling. The molecular design of Y5 underpins the dual PDT-PTT functionality of the polymer PYT. Under 808 nm laser irradiation, PYT NPs elevated the solution temperature from 26.1 °C to 69.1 °C while concurrently inducing a rapid decline in the absorption intensity of the ROS indicator 1,3-diphenylisobenzofuran (DPBF), confirming robust photothermal and photodynamic performance. Conventional monotherapy often fails to eradicate tumor cells completely, leaving residual cells prone to recurrence. In contrast, the combined PDT-PTT effects of PYT enable comprehensive tumor tissue disruption, minimizing residual cancer cells. In vivo antitumor experiments demonstrated that tumors treated with “PYT NPs + NIR” were completely eradicated within 12 days, effectively reducing the likelihood of tumor recurrence. This synergistic approach highlights the translational potential of dual-modal nanoplatforms in overcoming therapeutic limitations associated with tumor heterogeneity and treatment resistance [94] (Figure 5b).

### 3.2. Photodynamic Immunotherapy

Metastatic cancers, characterized by malignant cell dissemination to distant organs, necessitate therapeutic strategies that integrate primary tumor eradication with systemic immune activation [96,97,98]. PDT can induce immunogenic cell death (ICD) and tumor antigen release. The underlying mechanism involves cytotoxic ROS generated during PDT, which damage or necrotize tumor cells, triggering the release of damage-associated molecular patterns (DAMPs) such as calreticulin (CRT), high-mobility group box 1 (HMGB1), adenosine triphosphate (ATP), and heat shock proteins (HSPs). These DAMPs are recognized by dendritic cells (DCs), promoting DC maturation, antigen presentation, and pro-inflammatory cytokine secretion, thereby driving CD8⁺ T cell differentiation into cytotoxic T lymphocytes (CTLs) and initiating antitumor immunity [99]. However, the immunotherapeutic efficacy of PDT alone is often constrained by the immunosuppressive TME [100,101]. To address these limitations, the macromolecular backbone of CPs can be functionalized to load immunomodulators for reshaping the immunosuppressive TME. Additionally, side-chain modification sites enable conjugation with immunotherapeutic agents, synergistically enhancing ICD intensity, amplifying local immune responses, and deepening systemic immunity. This multilevel “in situ killing–immune priming–distal suppression” therapeutic paradigm not only eradicates primary tumors efficiently but also suppresses metastatic recurrence through immune memory effects, offering a novel approach to combat metastatic cancers.

**Figure 6:**
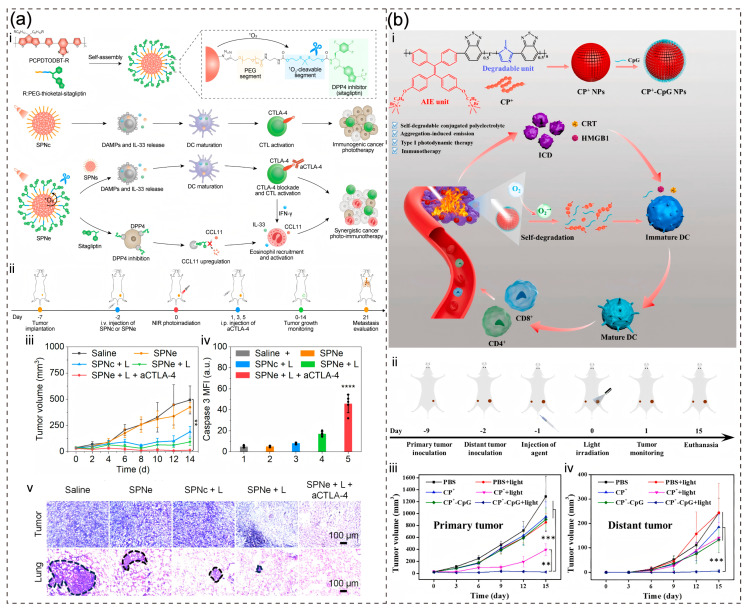
(**a**) (**i**) Schematic illustration of the synthetic routes, chemical structure of eosinophil-activating semiconducting polymeric nanoparticles (SPNe), and their mediated activatable cancer photo-immunotherapy. (**ii**) Schematic illustration of the experiment timeline for SPNe-mediated cancer photoimmunotherapy on 4T1 tumor-bearing mice. (**iii**) Tumor growth profiles of 4T1 tumorbearing mice after different treatments (n = 5). SPNe + L + aCTLA-4 versus saline: ** *p* < 0.01. (**iv**) Caspase 3 MFIs of the tumors from 4T1 tumor-bearing mice after different treatments (n = 5). SPNe + L + aCTLA-4 versus saline: **** *p* < 0.0001. (**v**) H&E staining images of tumor and lung tissues. The dotted circle indicates tumor metastatic niches [102]. Copyright © 2024 Wiley. VCH GmbH. (**b**) (**i**) Schematic illustration of the preparation procedure and the mechanism of CP^+^-CpG NPs on the synergistic treatment of PDT and immunotherapy. (**ii**) Schematic of the time schedule for primary and distant tumor inoculation, injection of the agent, light irradiation, tumor monitoring, and euthanasia. (**iii**) Primary tumor growth curves with the different treatment groups at different times. (**iv**) Distant tumor growth curves with the different treatment groups at different times. Data were shown as mean ± SD (n = 5), ** *p* < 0.01, and *** *p* < 0.001 determined by Student’s t-test [103]. Copyright © 2022 Published by Elsevier B.V.

Zhang et al. developed eosinophil-activating SPNe nanoparticles by covalently linking sitagliptin (a dipeptidyl peptidase 4 (DPP4) inhibitor) and polyethylene glycol (PEG) via a ^1^O_2_-cleavable thioketal linker, followed by conjugation with the photosensitive polymer PCPDTODBT. Immunofluorescence staining for ICD revealed that tumor cells treated with SPNe under NIR irradiation exhibited an HMGB1 mean fluorescence intensity (MFI) of 20.4% compared to non-irradiated controls, confirming SPNe-mediated ^1^O₂ generation for tumor cell killing and ICD induction. Upon light exposure, ^1^O₂ cleaved the thioketal linker to release sitagliptin, which inhibited DPP4 activity, suppressed DPP4-mediated degradation of the eosinophil-recruiting chemokine CCL11, and upregulated tumor-infiltrating eosinophils. ELISA results demonstrated a 6.5-fold increase in IL-33 (an extracellular eosinophil-activating cytokine), a 37.3% reduction in DPP4 activity, and a 1.4-fold elevation in CCL11 levels compared to PBS-treated controls. Further combination with anti-CTLA-4 antibodies enhanced T-cell activation and function, increasing IFN-γ secretion and eosinophil activity. This multilevel synergy significantly elevated populations of CD3⁺CD8⁺ T cells, Granzyme B⁺ CTLs, and IFN-γ⁺ CTLs in tumors, effectively suppressing tumor growth and metastasis. In 4T1 tumor-bearing mice, H&E staining of lung tissues showed minimal metastatic foci in the SPNe/aCTLA-4 + NIR group, while PBS and control groups exhibited over 10 metastatic nodules, validating the combinatorial efficacy of SPNe-mediated photoimmunotherapy and checkpoint blockade in suppressing metastasis [102] (Figure 6a).

Huang et al. synthesized a self-degradable conjugated polyelectrolyte (CP⁺) containing imidazole units, which electrostatically adsorbed negatively charged cytosine-phosphate-guanine (CpG) oligonucleotides to form CP⁺-CpG NPs for photodynamic immunotherapy. CpG, a single-stranded synthetic DNA, acts as an immune adjuvant but faces challenges in cellular internalization due to its hydrophobic structure and rapid enzymatic degradation in vivo. The positively charged CP⁺ facilitated CpG delivery into cells through electrostatic interactions. Upon light irradiation, CP⁺ generated •O_2_^−^, triggering its degradation and CpG release. In vitro, drug release assays confirmed significant CpG liberation post-irradiation. Immunofluorescence staining for ICD revealed enhanced CRT and HMGB1 signals in the “CP⁺-CpG + Light” group, confirming PDT-induced ICD. Flow cytometry demonstrated marked increases in CD80⁺CD86⁺ DCs, CD3⁺CD4⁺ T cells, and CD3⁺CD8⁺ T cells in the “CP⁺-CpG + Light” group compared to the controls. In a bilateral tumor model, untreated distal tumors exhibited growth suppression when the primary tumor received “CP⁺-CpG + Light” therapy, accompanied by elevated IFN-γ, IL-6, and TNF-α levels in tumor tissues. These results validate that CP⁺-CpG-mediated photodynamic immunotherapy activates antitumor immunity, effectively inhibiting both primary and distant tumor growth [103] (Figure 6b).

### 3.3. Chemotherapy-PDT

Traditional chemotherapy targets rapidly proliferating tumor cells through systemic drug administration, yet its clinical application faces significant challenges. A primary issue is drug resistance, whereby cancer cells develop adaptive mechanisms over time, such as drug efflux pumps and enhanced DNA repair, to evade chemotherapeutic effects [104]. Additionally, chemotherapy is notorious for systemic side effects. By indiscriminately affecting all rapidly dividing cells, it damages healthy tissues, leading to adverse reactions such as alopecia, nausea, fatigue, and immunosuppression, which severely compromise patients’ quality of life [105].

To enhance its chemotherapeutic efficacy, diverse strategies have been developed to optimize drug delivery. Among these, CP-based PDT represents a promising approach. The synergistic combination of chemotherapy and PDT significantly improves antitumor outcomes through multidimensional mechanisms. First, the ROS generated during PDT directly disrupts cancer cell membrane integrity, enhancing permeability and facilitating chemotherapeutic drug influx into tumor cells [106]. Second, while chemotherapeutic agents primarily target DNA synthesis or microtubule function during specific cell cycle phases, PDT-induced oxidative stress triggers cell death in a cycle-independent manner. These complementary mechanisms enable multi-pathway attacks on cancer cells [107,108]. More importantly, as a light-activated therapeutic modality, PDT exhibits spatiotemporal controllability. By precisely focusing a light source of specific wavelengths on the tumor region, the localized activation of photosensitizers to generate ROS minimizes damage to healthy tissues. The integration of chemotherapeutic drugs with CPs can reduce off-target toxicity caused by drug leakage, offering an innovative “high-efficacy–low-toxicity” strategy for cancer treatment.

**Figure 7:**
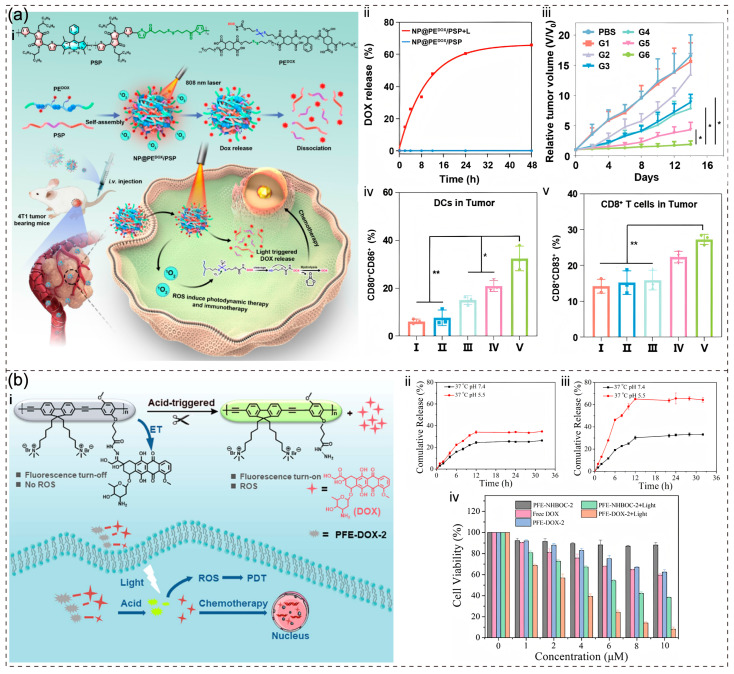
(**a**) (**i**) Schematic illustration of the design of self-sacrificially degradable NIR-II theranostic nanoparticles NP@PE^DOX^/PSP for photodynamic immunotherapy and Photoacoustic Computed Tomography. (**ii**) Cumulative DOX release upon light irradiation quantified by high-performance liquid chromatography. (**iii**) Data are represented as mean ± SD (* *p* < 0.05 vs. the control group, n = 5, two-way ANOVA test). Quantitative analysis of CD80^+^CD86^+^ DCs gated on CD11c^+^ tumor cells (**iv**) and of CD8^+^ and CD4^+^ T cells gated on CD3^+^ tumor cells (**v**) [109]. Copyright © 2022 Wiley. VCH GmbH. (**b**) (**i**) Schematic diagram of pH-responsive drug release and the mechanism of synergistic chemo-/PDT antitumor therapy. DOX release curves of (**ii**) PFE-DOX-1 and (**iii**) PFE-DOX-2 in PBS buffer with different pH values. (**iv**) cytotoxicity of PFE-NHBOC-2, PFE-DOX-2 and free DOX against MCF-7 cells without or with light irradiation (25 mW cm^−2^) for 30 min. All data are represented as mean ± SD (* *p* < 0.05, ** *p* < 0.005 vs. the control group, n = 5, one-way ANOVA test) [110]. Copyright 2022, MDPI.

Tang et al. constructed amphiphilic polyester PE^DOX^ loaded with doxorubicin (DOX), which co-assembled with a self-degradable conjugated polymer PSP to form nanoparticles (NP@PE^DOX^/PSP). Under 808 nm laser irradiation, ROS generated by PSP cleaved thioketal linkages in PEDOX, enabling rapid DOX release for chemotherapy. This light-triggered drug delivery mechanism ensured precise chemotherapeutic activation at tumor sites, enhancing intratumoral drug concentration while minimizing systemic toxicity. In vitro drug release assays demonstrated irradiation time-dependent DOX release from NP@PE^DOX^/PSP, whereas negligible release occurred without irradiation. As previously described, PDT-induced ICDs can convert immunologically “cold” tumors into “hot” ones, sensitizing chemotherapy-resistant cells to immune recognition and attack. Combining chemotherapy with PDT (“NP@PE^DOX^/PSP + L” group) significantly enhanced cytotoxicity against 4T1 and A549 cancer cells, with growth inhibition rates surpassing those of monotherapy groups. Furthermore, this combination therapy markedly increased the proportion of mature dendritic cells (CD80⁺CD86⁺) and CD8⁺ T cell infiltration in tumors [109] (Figure 7a).

Zhang et al. covalently conjugated DOX to water-soluble CPs via acid-sensitive hydrazone bonds, creating a pH-responsive drug delivery system (PFE-DOX-2) for chemo-photodynamic synergy. This design minimized systemic drug exposure and improved stability under physiological conditions (pH 7.4). In vitro release assays revealed 32% DOX release at pH 7.4, which surged to 66% under acidic tumor-mimicking conditions (pH 5.5). Cellular experiments demonstrated superior tumor-killing efficacy in the “PFE-DOX-2 + L” group compared to monotherapy groups, further validating the potent synergistic effects of this dual-modal strategy [110] (Figure 7b).

### 3.4. Chemiluminescence-Driven Light Source-Free PDT

PDT relies on external light sources to activate photosensitizers to generate ROS and kill tumor cells. However, in the treatment of deep-seated tumors, the limited penetration depth of external light impedes its delivery to the tumor site, compromising therapeutic efficacy. Additionally, the inability of external light to precisely target complex lesions risks damaging healthy tissues [111]. Chemiluminescence (CL), a phenomenon where chemical reactions generate light, provides an effective alternative excitation source for PDT [112]. The mechanism involves chemical excitation that elevates a substance from its ground state to an excited state, followed by energy release in the form of light as the excited state returns to the ground state. A rationally designed chemical reaction system can localize CL at specific pathological sites. This intrinsic light generation, independent of external irradiation, positions CL as a promising energy source for PDT.

**Figure 8:**
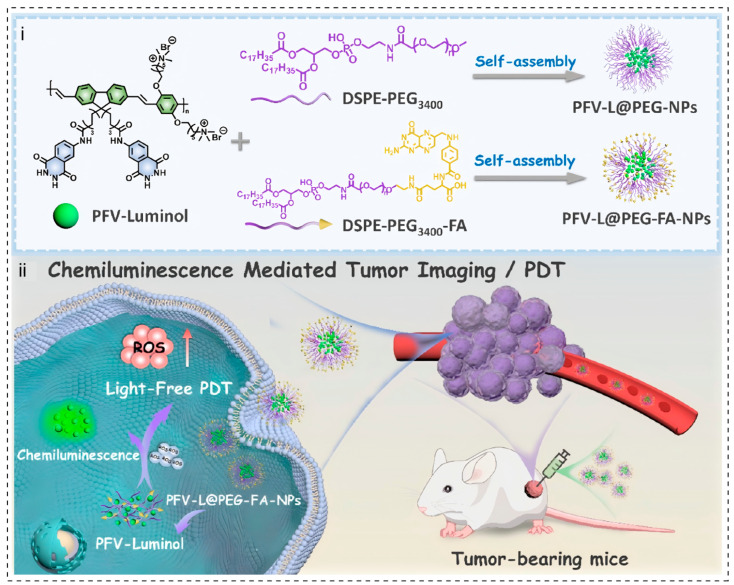
(**i**) Preparation of chemiluminescent nanoparticles PFV-L@PEG-NPs and PFV-L@PEG-FA-NPs. (**ii**) Chemiluminescence-mediated tumor imaging/PDT [113]. Copyright © 2024, American Chemical Society.

Li et al. successfully synthesized PFV-Luminol nanoparticles with a CL system by covalently conjugating isoluminol moieties to the side chains of PFV (a conjugated polymer) via amide bonds. In solution, PFV-Luminol exhibited a maximum absorption peak at 403 nm with broad absorption spanning 300–500 nm and an emission peak at 490 nm. The CL emission peak of isoluminol at 408 nm significantly overlapped with the absorption spectrum of PFV-Luminol, enabling efficient chemiluminescence resonance energy transfer (CRET). Isoluminol luminescence can be activated by ROS. In the absence of external light, PFV-Luminol solution treated with ROS displayed intense CL emission at 508 nm and green fluorescence emission from PFV. In contrast, a physical mixture of PFV and isoluminol showed minimal changes in CL emission and no detectable PFV fluorescence. These results confirm that the covalent conjugation strategy between isoluminol and PFV enables CL-driven activation of PFV. The covalent linkage drastically shortens the intermolecular distance, significantly enhancing CRET efficiency. Similarly, PFV-Luminol generates cytotoxic ROS via CRET only in the presence of ROS. The TME, typically characterized by high ROS concentrations, provides ideal conditions for CRET in PFV-Luminol, thereby activating PFV to produce ROS and enabling tumor-selective PDT. In vivo antitumor experiments demonstrated that PFV-Luminol significantly suppressed tumor growth without requiring external light, with tumor volumes markedly smaller than those in control groups. This CL-driven PDT strategy overcomes the limitations of external light dependency, offering a spatially precise and penetration-independent therapeutic approach for deep tumors. By harnessing endogenous ROS to trigger localized light emission and subsequent ROS amplification, PFV-Luminol exemplifies a self-sustaining system for targeted cancer therapy [113] (Figure 8).

### 3.5. Theranostics

**Figure 9:**
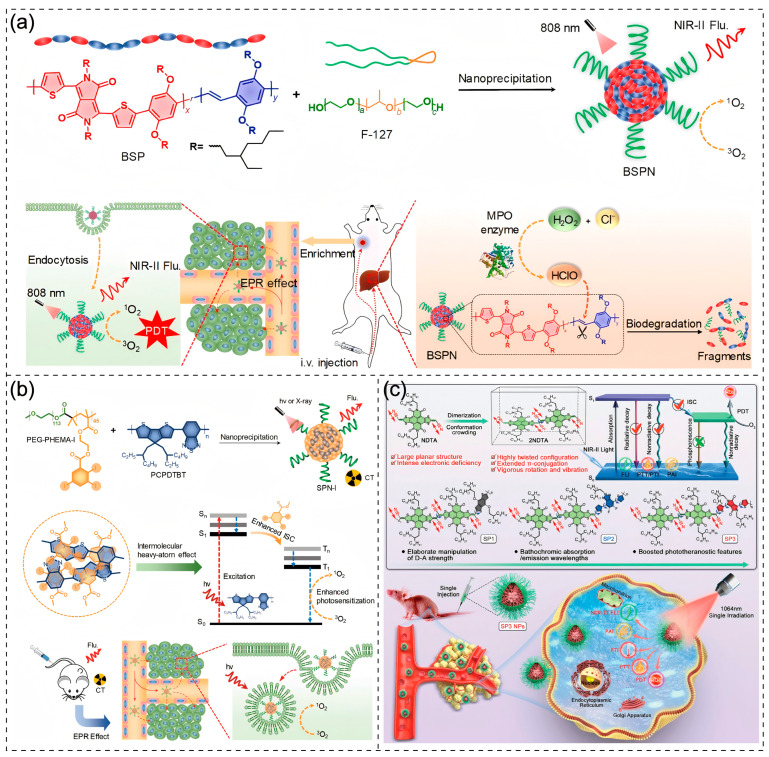
(**a**) Preparation schematic of BSPN and in vivo NIR-II imaging-guided PDT with biodegradation in liver [115]. Copyright © 2024, Tsinghua University Press. (**b**) Schematic illustrations of SPN-I preparation, iodine-enhanced photosensitization via heavy-atom effect, and in vivo CT/NIR-II dual-modal imaging-guided PDT [116]. Copyright © 2020 WILEY-VCH Verlag GmbH & Co. KGaA Weinheim. (**c**) Schematic illustration of molecular design and resulting high-performance NIR-II excitable semiconducting polymer with AIE characteristic for fluorescence-photoacoustic bimodal imaging-guided synergistic phototherapy [117]. Copyright © 2024 Wiley. VCH GmbH.

Conjugated polymers, with their unique optoelectronic properties, demonstrate immense potential in tumor theranostics. On the one hand, the photodynamic effect of CPs generates ROS through photosensitization, directly inducing tumor cell apoptosis for effective therapy [69,114]. On the other hand, their exceptional fluorescence characteristics enable high-sensitivity, high-resolution imaging, providing precise tumor localization. Gu et al. designed a NIR-II fluorescent imaging nanotheranostic system, BSPN50. The CPs in this system incorporate the strong electron-withdrawing group diketopyrrolopyrrole (DPP), which broadens the material’s absorption and emission wavelengths into the NIR-II region. In vivo imaging in mice revealed that under 808 nm laser excitation, BSPN50’s NIR-II fluorescence signal clearly visualized abdominal blood vessels, achieving a signal-to-background ratio (SBR) of 2.97 and a full width at half maximum (FWHM) of 0.23 mm, indicating superior imaging resolution. Following intravenous injection into tumor-bearing mice, the tumor fluorescence intensity increased fourfold at 6 h, demonstrating efficient tumor accumulation and laying the foundation for image-guided precision PDT [115] (Figure 9a).

Moreover, PDT can be integrated with multiple imaging modalities, such as X-ray Computed Tomography (CT) and Photoacoustic Imaging (PAI), to provide multidimensional tumor information for targeted therapy. Zhou et al. co-assembled NIR-emitting PCPDTBT with an iodine-grafted amphiphilic copolymer (PEG-PHEMA-I) to form iodine-rich SPN-based nanotheranostic (SPN-I) for CT-fluorescence dual-modal imaging-guided PDT. The iodine-endowed SPN-I, with a high X-ray attenuation coefficient, enables CT imaging. In vivo experiments showed that PCPDTBT-mediated fluorescence imaging sensitively localized tumors at early stages, while CT imaging delineated tumor morphology, size, and spatial relationships with surrounding tissues once SPN-I accumulated sufficiently [116] (Figure 9b). Li et al. constructed a NIR-II laser-triggered fluorescence-photoacoustic theranostic platform (SP3) by coupling two naphthalene diimides (NDIs)-fused 2-(1,3-dithiol-2-ylidene)acetonitriles (NDTA) units with DPP. The D-A structure of 2NDTA and DPP conferred SP3 with AIE characteristics and broad absorption in the NIR-II region. Under 1064 nm laser irradiation, SP3 in solution elevated temperatures to 79.2 °C, demonstrating exceptional photothermal conversion efficiency critical for photoacoustic signal generation. In vivo, SP3 enabled NIR-II fluorescence and PAI dual-modal imaging post-injection, with tumor fluorescence peaking at 24 h and PAI clearly outlining deep tumor margins. This “diagnosis–therapy” integrated design not only enhances therapeutic precision but also reduces operational complexity and risks associated with conventional separated diagnostic and therapeutic approaches [117] (Figure 9c).

### 3.6. CDT-PDT

CDT, an emerging tumor treatment strategy, exploits specific chemical conditions in the TME (e.g., weak acidity and elevated H_2_O_2_) to trigger Fenton or Fenton-like reactions, converting H_2_O_2_ into highly toxic •OH for selective cancer cell killing [118,119]. CDT requires catalysts such as iron or manganese ions. Its selectivity, driven by the biochemical differences between tumor and normal tissues, minimizes off-target damage. Unlike light-dependent PDT, CDT operates independently of external illumination, enabling therapeutic coverage in deep tumor regions inaccessible to PDT. The combination of PDT and CDT achieves a multidimensional therapeutic effect: PDT eradicates superficial tumors, while CDT penetrates residual lesions, enabling comprehensive tumor ablation [120,121]. Ding et al. developed a combined PDT-CDT therapeutic platform (SPN-oxy-Hb@RBCM) by self-assembling the photosensitizing polymer PCPDTBT with poly(styrene-co-maleic anhydride) (PSMA) to form semiconducting polymer nanoparticles (SPNs), followed by covalent conjugation of hemoglobin (Hb) via carbodiimide reaction. In this system, Hb serves as an oxygen carrier to replenish tumor oxygenation, thereby alleviating hypoxia and enhancing PDT efficacy. 

**Figure 10:**
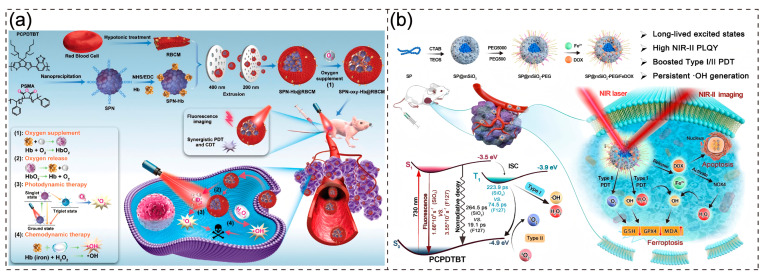
(**a**) Schematic Illustration for the Preparation of the SPN-oxy-Hb@RBCM Theranostic Nanoagent and Its Application in Fluorescence Imaging (FI)-Guided Chemo- and Photodynamic Therapy of Solid Hypoxic Tumors [122]. Copyright © 2021, American Chemical Society. (**b**) Schematic illustration of the synthetic process for the semiconducting polymer-based nanoplatform and its applications in phototheranostics [123]. Copyright 2024, The Royal Society of Chemistry.

Simultaneously, the iron in Hb can catalyze a Fenton-like reaction with excess hydrogen peroxide in the tumor microenvironment, generating cytotoxic •OH for CDT. In vivo antitumor experiments demonstrated that the “SPN-oxy-Hb@RBCM + L” group (with laser irradiation) exhibited the most significant tumor growth inhibition, with markedly smaller tumor volumes compared to other treatment groups. These results confirm that the synergistic integration of CDT and PDT can simultaneously harness the advantages of both therapeutic modalities at the tumor site, enabling efficient tumor cell eradication while suppressing tumor growth and metastasis [122] (Figure 10a).

Notably, certain Type I photosensitizers can photogenerate H_2_O_2_, providing a substrate for CDT to amplify oxidative stress in the TME. Lu et al. encapsulated PCPDTBT (a Type I/II photosensitizers) within a mesoporous silica matrix and surface-adsorbed Fe^2+^ as a CDT catalyst. Under irradiation, PCPDTBT generated H_2_O_2_ via Type I PDT. Post-irradiation, Fe^2+^-loaded particles continued to degrade the •OH indicator 3,3′,5,5′-tetramethylbenzidine (TMB), while Fe^2+^-free controls showed no such activity. This confirms that photogenerated H_2_O_2_ synergizes with Fe^2+^ to sustain Fenton reactions, amplifying tumoricidal efficacy [123] (Figure 10b).

## 4. Summary and Outlook

PDT has emerged as a novel cancer treatment approach that demonstrates tremendous potential in oncology due to its advantages of minimal invasiveness, spatiotemporal precision, and low toxicity. With their unique π-conjugated structures and tunable photophysical properties, CPs have become ideal candidate materials for a new generation of highly efficient photosensitizers. This review systematically summarizes molecular design strategies to enhance the photodynamic performance of CPs, including constructing D-A structures, introducing AIE groups, utilizing heavy-atom effects, and designing hyperbranched topologies. These strategies significantly improve ROS generation efficiency by regulating excited-state behaviors. To address the therapeutic challenges posed by tumor heterogeneity and microenvironment complexity, we explore synergistic approaches combining CP-mediated PDT with other treatment modalities such as photothermal therapy, immunotherapy, and chemotherapy. These multimodal combination therapies can fully leverage the “1 + 1 > 2” synergistic effect, providing novel solutions to overcome the limitations of single-treatment approaches.

Future research should focus on several key directions: (1) developing NIR-II-responsive CP photosensitizers to address insufficient tissue penetration depth; (2) designing smart-responsive CPs capable of specific reactions to TEM factors like pH, enzymes, and ROS; (3) further investigating the interaction mechanisms between CPs and the immune system to optimize combined immuno-PDT treatment protocols; (4) establishing standardized biosafety evaluation systems for CPs to facilitate clinical translation; (5) integrating artificial intelligence technology to accelerate the rational design of high-performance CPs photosensitizers. With the continued convergence of materials science, nanotechnology, and biomedicine, CP-based multimodal precision treatment strategies are expected to provide innovative solutions for overcoming the major medical challenge of cancer.

## Data Availability

Not applicable.
